# Class Address

**Published:** 1872-04

**Authors:** T. H. Ferguson


AMERICAN JOURNAL

OF

DENTAL SCIENCE.

Vol. V. THIRD SERIES--APRIL, 1872. No. 12.

### ARTICLE I.

### *Class Address.*

Delivered at the Thirty-second Annual Commencement of the Baltimore College of Dental Surgery. By T. H. Ferguson, a Graduate.

I have been selected by the members of the Graduating Class to represent them upon this occasion. As the recipient of this honor, I am deeply sensible of the task imposed upon me in appearing before this large assemblage, and therefore at the outset bespeak your kind indulgence.

The joy that we feel at meeting so many of you to-night, is tinged with sorrow, for on to-morrow's dawn we must sever our connection from a scene which may never again come within the range of our vision. We came her strangers among you, and were met with that kindness and hospitality which has ever been and will continue to be characteristic of a genial, warm-hearted Southern people. And now when our sojourn among you is ended, let me assure you that these acts of yours will not be forgotten, but will be indelibly written upon the book of our memory where every day will turn the leaf to read them. It may seem vanity and presumption on my part, but a knowledge of its genuine worth and importance to this community prompts me to congratulate the citizens of Baltimore on the possession of a College of Dental Surgery. The benefits to be derived from this and kindred institutions redound largely to the progress of the dental art, and to the happiness and physical comfort of this nation. Let there be no abatement in that interest which, by your presence here to-night, I know you feel; for under your fostering care and your liberal patronage this noble institution will ever rest as now, upon the flood tide of prosperity.

Gentlemen of the Faculty, yours has been an arduous task, but patiently and faithfully have you performed your respective duties. The zeal you have evinced in a good cause has been untiring, and the efficiency which you have shown as lecturers and demonstrators will find its reward, let us hope, in the satisfactory work of those who have availed themselves of your excellent instruction. With such a Faculty the attentive student cannot be lacking in all the requisite theoretical knowledge, and by the pursuance of a little more liberal policy, thereby securing a more extended public patronage, those essential qualifications which ensure success, will never be wanting to the student at the early stage of his professional career. This Dental College, of which you are the head and front, is the pioneer institution of the land. Its reputation is not hedged in by the confines of our own native soil, but has almost become a household word in other countries, and even the fair sex of foreign climes knock at its doors for admission. With such a world wide fame who can doubt there remains for it a glorious destiny? May its star never set, but ever remain lustrous as the high fame of that great man its founder, whose memory neither slab nor monument, nothing but heaven *itself* can cover.

We who go forth to enter upon the practice of a profession the groundwork of which you have taught us, will give you the largest place in our recollections of the present, not only for the principles you have inculcated, not only for the useful knowledge imparted, but for the uniform kindness and courtesy you have extended to each one of us; those voluntary, exemplary acts which emanate only from true, large-hearted gentlemen.

Classmates, to you it remains for me to speak and my task is done. You have come here from the South and West for one common purpose; to lay the foundation of that knowledge on which your success in the profession you have adopted, depends. By your act of coming, and the purpose of your life exemplified in your studiousness and arduous labor of many months duration, as well as the crucial test of an examination to which you have been subjected as candidates for graduation, give unmistakable evidence to the world that you are fully alive to its stringent requirements and the difficulties attending the skilful practice of that profession; that you wisely chose to serve an apprenticeship at the work before grappling with the responsibilities which accompany all successful achievements in the dental art. It is not for us to disclaim the knowledge that there are some practitioners who have risen to the highest pinnacle of success and eminence, who were never within the walls of a dental institution; but we must not ignore the fact that the countless number of quacks—for their number is legion—which infest every community are likewise men who never saw and who never wished to see the interior of such an institution. While you find one self made individual, who, by long experience, patient study, and assiduous labor, has become noted as an eminent dentist, you will be thrown in competition with scores who neglected or cared not for the opportunities of college education, and entered upon their profession actuated solely by a love of gain and not by any desire to enoble the art of their selection. Such men are the bane of civilization, and their cheap work which so attracts the gullible is a stigma upon the profession and a curse upon a patronizing community. You to night have received your diplomas which entitle you to enter upon your chosen work, and to the recognition and encouragement of the profession. This is the most trying period of your life. You know the difficulties which environ you, the responsibilities which cluster around your pathway, as "thick as stars on the brow of night," and it requires all your fortitude of character and stability of purpose to enter upon the struggle. This is the period to form a resolution which is to govern your future exertions, now it is that you should map out your plan of future action. Let your aim in life be *success* in every undertaking, and to approximate, if you cannot reach, a standard of perfection in all you undertake.

To attain success you must *work.* Patient, unremitting labor, is the only agent through which you can reach its attainment. Genius is vouchsafed to but few, and what we call its brilliant scintillations are but the magnificent results of searching thought and unflagging toil.

Take a true record of the lives of great men who have lived; who seemed to their contemporaries the very embodiment of genius, and you will find that it was by the most sedulous labor that they acquired their greatness. The gifted Sheridan gained the greatest reputation among the men of his time as an impromptu wit and orator; but the biographer has shown that his brilliant sallies of wit and humor were prepared before hand, and through the tact of the great man conversation was shaped for their easy and fitting introduction. What was true of his dazzling wit in the social circle, applied with equal verity to his eloquence in the halls of government.

Alexander Hamilton once remarked in substance, "Men give me credit for the possession of talent and genius, but I have never accomplished anything yet, except by the most arduous labor."

With such examples before you, Classmates, you must not rely *wholly* upon your standing capital of genius for success in life. The first requisits to ensure success is to make oneself *known.* This has been done by social intercourse, by means of the daily paper, or by sundry other legitimate methods. There is a great prejudice on the part of old established dentists against the insertion of professional cards in the newspaper. They resorted to this practice themselves at the commencement of their career, and after they have grown rich and gray in service they see the error of early youth, and seek atonement for its commission, not by giving up their gains, the result of that early indiscretion, but by denouncing it as one of the unfair methods of making one's business known, which belongs exclusively to charlatanism.

That quacks resort to advertising columns largely it is not for me to deny, but it is folly to maintain that they alone have such recourse. It is legitimate, for it aids in a good work by promoting the welfare of the newspaper. All trades or professions are mutually dependent on each other, and each must render its assistance to the other to ensure their several and common prosperity. Therefore do not let the daily paper go down and your business go up, because you entertain a prejudice against the laudable practice of contributing to the support of that paper. They tell us ability and merit will always make their way regardless of surrounding circumstances. Believe it not. Such thoughts are antediluvian. These admirable qualities did once upon a time find a fitting reward in an appreciative public, *but it is not so now*. *Now* cheek is the autocrat of the day. Cheek is at the front and carries away the golden honors, while modest merit in the background sits "like patience on a monument," and quietly looks on.

Gentlemen go in to win. Let *success* be your motto. Emblazon it upon your banner which you unfurl to the public gaze, and be sure you keep at least one eye always upon it. If people cannot see your modest door-plate, rear a sign colossal in its proportions, and if still the public eye is blind to things aloft and remains solidly fixed upon the bricks beneath, *then* plant a marble tooth in front of your office door and success is inevitable—your fortune is made.

Our emotional natures are stirred by a two-fold influence to-night. Joy and sorrow are ultimately blended and ac h is battling for supremacy on this occasion which precedes the morrow of our separation. We are glad that the time draws near which is to mark our welcome home. Already we think we feel the imprint of a fond sister's kiss, we receive a mother's prayerful welcome, a father's cordial greeting.

But the home picture is withdrawn and another held up to our view in the scene before us. Here we have our friends in Baltimore, our Professors and each other. Strangers at our first meeting, but as time grew apace, by a common pursuit thrown in daily, almost hourly contact, our friendships have strengthened, and our hearts grow sad as the hour approaches which is to point the moment of our departure. Let our frindships here which we have formed among ourselves and among our acquaintances ever be cherished as the fondest recollections of our college career.

With these few, brief words, Ladies and Gentlemen, Gentlemen of the Faculty, Classmates, one, *all*! I bid you an earnest, an affectionate *farewell!*

---

